# Solvent-Free Addition of Indole to Aldehydes: Unexpected Synthesis of Novel 1-[1-(1*H*-Indol-3-yl) Alkyl]-1*H*-Indoles and Preliminary Evaluation of Their Cytotoxicity in Hepatocarcinoma Cells

**DOI:** 10.3390/molecules22101747

**Published:** 2017-10-17

**Authors:** Graziella Tocco, Gloria Zedda, Mariano Casu, Gabriella Simbula, Michela Begala

**Affiliations:** 1 Department of Life and Environmental Sciences, Unit of Drug Sciences, University of Cagliari, via Ospedale 72, 09124 Cagliari, Italy; michelabegala@unica.it; 2 Merck Millipore, 39 Route Industrielle de la Hardt, 67120 Molsheim, France; gloria.zedda@merckgroup.com; 3 Department of Physics, University of Cagliari, 09042 Monserrato CA, Italy; mcasu@unica.it; 4 Department of Biomedical Science, Oncology and Molecular Pathology Unit, University of Cagliari, 09124 Cagliari, Italy; gsimbula@unica.it

**Keywords:** solvent-free reaction, 1-[1-(1*H*-indol-3-yl) alkyl]-1*H*-indoles, hepatocarcinoma, cytotoxicity

## Abstract

New 1-[1-(1*H*-indol-3-yl) alkyl]-1*H*-indoles, surprisingly, have been obtained from the addition of indole to a variety of aldehydes under neat conditions. CaO, present in excess, was fundamental for carrying out the reaction with paraformaldehyde. Under the same reaction conditions, aromatic and heteroaromatic aldehydes afforded only classical bis (indolyl) aryl indoles. In this paper, the role of CaO, together with the regiochemistry and the mechanism of the reaction, are discussed in detail. The effect of some selected 3,3′- and 1,3′-diindolyl methane derivatives on cell proliferation of the hepatoma cell line FaO was also evaluated.

## 1. Introduction

Indole is one of the most versatile heterocyclic nuclei, identified as a pharmacophore in a large number of natural and synthetic biologically active molecules [1]. Due to its electron-rich character, indole promptly reacts with hard and soft electrophiles; thus, it is fair to consider it a privileged structure, quaintly referred to as the ‘lord of the rings‘ [2].

Today, considerable attention is focused on a number of 3,3′-diindolyl methanes (or bis (indolyl) methanes) (3,3′-BIMs), structurally dimers of the dietary component indole-3-carbinol (**I3C**), with whom they share the same ability to suppress proliferation and induce apoptosis in various cancer cells [3,4,5]. In view of their importance, many methods have been reported for the synthesis of 3,3′-BIM compounds, using a multitude of catalysts such as protic or Lewis acids, I_2_, zeolite, K-10 clay, ZnO, water, gold(I)-complexes, oxalic acid dehydrate, cobalt nanocatalyst, nanoparticles, graphene oxide [6,7,8,9,10,11,12,13,14,15,16,17] and various reaction conditions (e.g., ionic liquids, flow chemistry, etc.) [18,19]. However, to the best of our knowledge, reports on the preparation of 1,3′-diindolyl methane derivatives are rare [20,21,22,23].

**Scheme 1:**
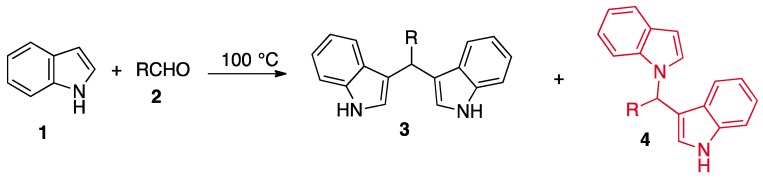
Reaction between indole **1** and aliphatic aldehydes **2**.

We report herein the first example of the synthesis of racemic 1-[1-(1*H*-indol-3-yl) alkyl]-1*H*-indoles through a direct solvent-free Mannich-type addition reaction of indole and aliphatic aldehydes (Scheme 1).

Interestingly, several natural [24] and synthetic 3,3′-BIMs have shown important anti-cancer properties and demonstrated the capacity to sensitize cancer cells to apoptosis by signaling various proapoptotic genes and proteins [25,26,27]. In this regard, we present a preliminarily evaluation of the effect of these novel 3,3′- and 1,3′-BIM derivatives on the growth of the hepatoma cell line FaO.

## 2. Results and Discussion

**Scheme 2:**
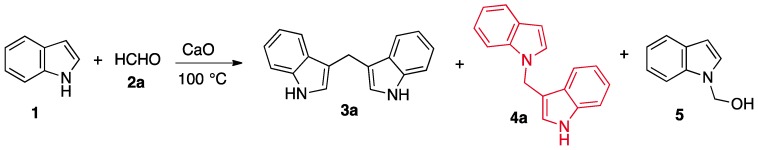
Reaction between indole **1** and formaldehyde **2a**.

In the quest to develop a simple and eco-friendly protocol to 3,3′-BIM derivatives, we unexpectedly observed, from the very beginning of our study, that, when the reaction was carried out with formaldehyde or aliphatic aldehydes, both 3,3′- and 1,3′-bisindolyl methane (3,3′- and 1,3′-BIM) derivatives were obtained. Conversely, aromatic aldehydes gave only classical 3,3′-BIMs. To understand these unusual results, the reaction between indole **1**, and formaldehyde **2a**, derived from paraformaldehyde, was first investigated (Scheme 2).

**Figure 1:**
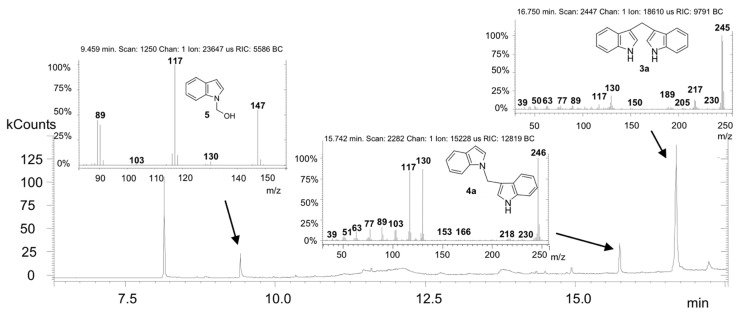
Gas chromatogram (GC) and mass spectra of compounds **3a**, **4a** and **5**.

Surprisingly, apart from the expected 3,3′-diindolyl methane **3a**, we observed, as shown in Scheme 2, the formation of indole-1-carbinol **5** [28] and 1-[1-(1*H*-indol-3-yl) methyl]-1*H*-indole **4a**, which was fully characterized via mass spectrometry (MS) and nuclear magnetic resonance (NMR) spectroscopy experiments (spectra available in Supplementary Materials). The best results were achieved at 100 °C, and also observed an improvement in the yields when an excess of calcium oxide (CaO) was used. The reaction did not take place at all at room temperature, both with or without CaO. Similarly, when the operative temperature was fixed at 60 °C, traces of both isomers were detectable. Thus, it was speculated that CaO, which has recently been shown to catalyse some Mannich reactions for the preparation of lariat ethers [29], might affect our experiments. Hence, the role of CaO was explored, noticing that, when it was used in stoichiometric amounts or in large excess, the reaction seemed to proceed smoothly. Use of CaO in a catalytic amount (10 mol %), did not seem to produce any effect. Reasonably, the temperature and CaO might have a synergic effect in the reaction activation. We can plausibly assume that, in these conditions, the paraformaldehyde is rapidly converted to free formaldehyde and the traces of formic acid produced during prolonged heating are rapidly neutralized by CaO. Following the experiment by means of gas chromatography–mass spectrometry (GC–MS) analysis (Figure 1), we also observed, from the very beginning of the reaction and only in the presence of CaO, the formation of the indole-1-carbinol **5**, which is usually obtained in a strong basic condition [30,31].

We postulate that, in our experimental conditions, CaO induces a partial N–H proton abstraction and, despite the modest ionic character of the N–Ca bond, formaldehyde is so reactive as to undergo a nucleophilic attack, generating indole-1-carbinol **5** [28]. As a matter of fact, when DMSO was used as polar aprotic solvent, the reaction afforded 1-[1-(1*H*-indol-3-yl) methyl]-1*H*-indole **4a** as a major product in a high regioselective manner.

In addition, an interesting yield improvement, especially for isomer **4a**, was noticed when KOH was used instead of CaO. A possible explanation, apart from the stronger electropositivity of K^+^, might be that indole-1-carbinol **5**, when heated in the presence of KOH, decomposes to regenerate indole and HCHO [30] that react again to afford both 3,3′- and 1,3′-BIM isomers (Table 1).

**Scheme 3:**
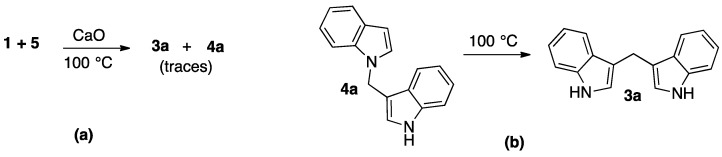
Investigation of the role of indole-1-carbinol **5** in the formation of compounds **3a** and **4a**.

To gain a better comprehension of the reaction mechanism, we examined the possible role of compound **5**. It did not demonstrate being a real intermediate in the formation of 1-[1-(1*H*-indol-3-yl)methyl]-1*H*-indole **4a**. In fact, when **5** could react with indole **1** and CaO, only traces of the two isomers were detected (Scheme 3a). Interestingly, the classical bis (indolyl) methane **3a**, could derive from a thermal-induced isomerisation of **4a** (Scheme 3b) [20].

With the perception that the reaction, if successful with formaldehyde, would allow similar 1,3′-diindolyl isomers when different carbonyl substrates were used, some other exploratory reactions with diverse ketones and aldehydes were carried out (Table 2).

It was immediately evident that the reaction was highly chemoselective for aldehydes; in fact, ketones such as 2-octanone, 2-hexanone, 4′-methylacetophenone and 1-(*p*-methoxyphenyl)-2-propanone did not react at all. Besides, only aliphatic aldehydes afforded both 3,3′- and 1,3′-isomers, while aromatic and heteroaromatic ones produced only bis (indolyl) aryl methanes. Interestingly, the reactions efficiently proceeded under neat conditions [35] and the yields seemed to be positively affected by the absence of CaO, which demonstrated its importance mainly for the paraformaldehyde activation. Remarkably, the reactions almost did not occur or took place very slowly, with or without CaO, when a solvent (toluene, CH_3_CN, CHCl_3_, THF, DMF) was employed [35].

**Scheme 4:**
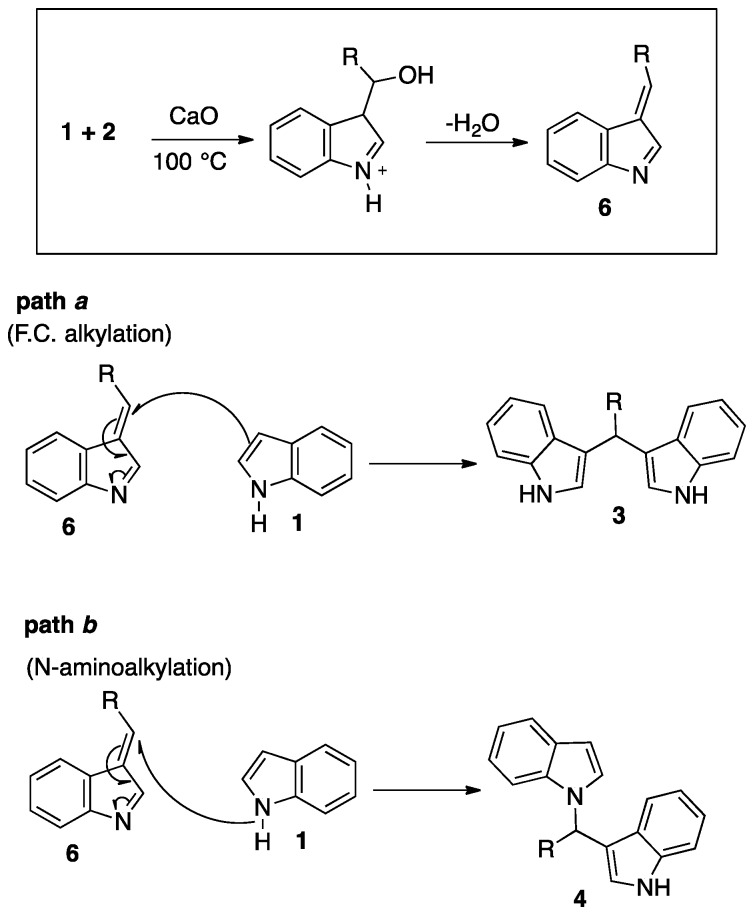
Proposed reaction mechanism.

Mechanistically, we presume (Scheme 4) that two reaction pathways can be conceived for the formation of 3,3′- and 1,3′-BIMs isomers. Both routes share the same well-known 2-azafulvene intermediate [20,21,22,23,36,37] which may add to nucleophilic species. Specifically, if the path a could be merely assumed as a Friedel–Crafts alkylation, the path b is presumably a Mannich-type *N*-aminoalkylation. In this respect, the different reactivity of aliphatic and aromatic aldehydes seems plausible given their intrinsically diverse steric and electronic features, and associated with the modest nucleophilicity of the indole nitrogen.

**Figure 2:**
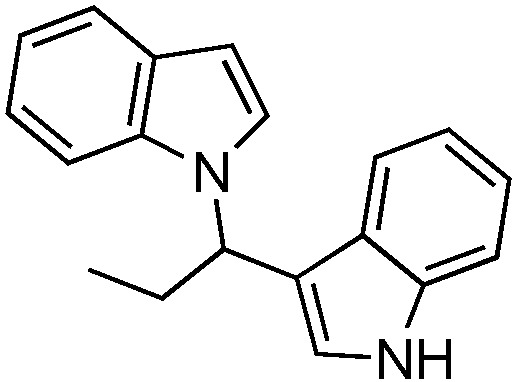
Electron-impact mass spectrometry (EI-MS) spectra of compounds **4b** and **3b**.

Electron-impact (EI) mass spectra allowed us to characterize and differentiate both isomers easily (Figure 2).

In fact, in the mass spectra of all 1,3′-diindolyl alkane isomers, the formation of the [M-116]^+^ ion, presumably due to N–C bond cleavage, is the most favored fragmentation process (RA % 100), while in the case of 3,3′-isomers, the formation of [M-116]^+^ ion (RA % 5), related to the rupture of the more stable C–C bond, is suppressed in favor of *m*/*z* 245 ion (RA % 100), generated by the radical loss of R substituent. MS/MS experiments are now in progress to investigate the most characteristic fragmentation pathways. NMR analysis included ^1^H, ^13^C, COSY, gHSQC, gHMQC and ROESY (spectra available in Supplementary Materials), and confirmed the structure of 1,3′-diindolyl alkane isomers.

**Figure 3:**
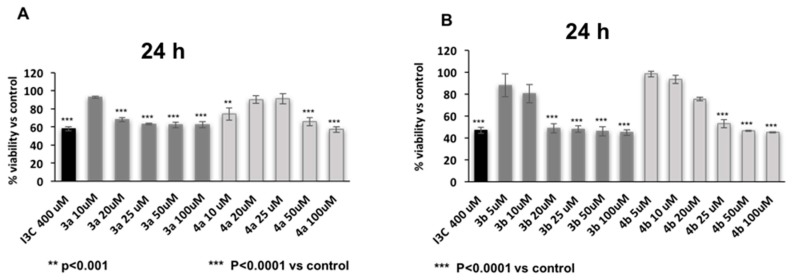
Effect of 400 µM **I3C** and increasing concentrations of the indole derivatives (**A**) **3a**, **4a** and (**B**) **3b**, **4b** on FaO cell viability, determined by neutral red uptake (NRU) assay after 24 h of treatment. Data are expressed as percentage of viable cells assessed by neutral red.

Recent studies have reported on the pleiotropic protective properties on the chronic liver injuries steatohepatitis [38] and hepatocarcinoma [39,40], and on the multiple anti-tumour activities, including the apoptotic, anti-proliferative and anti-angiogenetic effects [24], of 3,3′-bisindolylmethane, the parent compound of 3,3′-BIMs and one of the most abundant dietary compounds derived from *Brassica*-genus vegetables. Hence, we decided to evaluate the effect of our novel 1,3′ and 3,3′-BIMs on cell proliferation of the rat hepatoma cell line FaO by comparing their effects to those induced by 3,3′-bisindolylmethane **3a** and indole-3-carbinol (**I3C**), the natural active precursor of 3,3′-bisindolylmethane. In experiments, carried out on a selection of compounds, FaO cells were treated with increasing concentrations of **3b**, **4a** and **4b** as well as 400 µM of **I3C** for 24 h. As shown in Figure 3, the hepatoma cells were highly susceptible to the anti-proliferative effect of these BIMs. In particular, compounds **4a**, **3b** and **4b** exhibited a concentration-dependent growth inhibitory effect in FaO cells similar to that observed after treatment with the well-characterized BIM **3a** [38,39].

More importantly, our novel derivatives, as well as **3a**, were 20- to 4-fold more potent than **I3C** in suppressing the viability of FaO cells with an IC_50_ value ranging from 50–100 µM after treatment with **4a** and from 20–100 µM and 25–100 µM after treatment with **3b** and **4b**, respectively. It was noteworthy that all the tested BIMs induced a significant inhibition of growth of hepatoma cells at lower concentrations than **I3C**. Furthermore, **3b** was more effective in inducing loss of viability at very low doses.

## 3. Materials and Methods

### 3.1. Chemistry

All starting materials were purchased from commercial sources (Sigma-Aldrich, Milan, Italy) and AlfaAesar Thermo Fisher Scientific, Karlsruhe, Germany with purity ≥98%) and used without further purification. Reaction progress was monitored by thin layer chromatography (TLC) using Aldrich silica gel 60 F254 (0.25 mm) with detection by ultra-violet (UV) light. Chromatography was performed using Aldrich silica gel (60–120) mesh size with freshly distilled solvents.

^1^H- and ^13^C-NMR were recorded by Varian 400 and 500 MHz (Varian Medical Systems, Palo Alto, CA, USA) using tetramethylsilane as the internal standard. Microanalysis for carbon, nitrogen and hydrogen (CHN) were performed by a Carlo Erba 1106 Elemental Analyzer (Milan, Italy).

GC–MS: Low resolution mass spectra were carried out on a Saturn 2000 ion-trap coupled with a Varian 3800 gas chromatograph (Varian, Walnut Creek, CA, USA) operating under EI conditions (electron energy 70 eV, emission current 20 μA, ion-trap temperature 200 °C, manifold temperature 80 °C, automatic gain control (AGC) target 21,000) with the ion trap operating in scan mode (scan range from *m*/*z* 40–400 at a scan rate of 1 scan·s^−1^). Aliquot of 1 μL of solutions 1.0 × 10^−5^ M in chloroform have been introduced into the gas chromatographer inlet. A Varian factor four low-bleed/MS capillary column (VF-5ms 30 m, 0.25 mm i.d., 0.25 µm film thickness) (Varian) was used. The oven temperature was programmed from 150 °C (held for 2 min) to 310 °C at 30 °C/min (held for 2 min). The temperature was then ramped up to 350 °C at 20 °C/min. The transfer line was maintained at 250 °C and the injector port 30/1 split) at 270 °C.

High-resolution mass spectra were obtained from the Department of Environmental Health Sciences Mass Spectrometry Laboratory of Mario Negri Institute for Pharmacological Research, Milan. High-resolution mass spectrometry (HRMS) experiments were carried out on a Finnigan LTQ hybrid ion trap/orbitrap.

### 3.2. Experimental Procedures

#### Synthesis of 1-[1-(1*H*-indol-3-yl) methyl]-1*H*-indole (**4a**)

Indole **1** (8.54 mmol), paraformaldehyde (10.25 mmol) and CaO (145.18 mmol) were blended with a magnetic stirrer for the period indicated (TLC) at 100 °C. After reaction, the crude mixture was extracted with CH_2_Cl_2_ and the obtained extract was passed through celite. The concentrated filtrate was flash chromatographed (hexane/ethyl acetate 3/1) on silica gel, obtaining the desired product.

#### Typical procedure for 1-[1-(1*H*-indol-3-yl) alkyl]-1*H*-indoles (**4b**–**e**)

A mixture of indole **1** (8.54 mmol) and the appropriate aldehyde (10.25 mmol) was stirred for the period indicated (TLC) at 100 °C. After reaction, the crude mixture was flash chromatographed (hexane/ethyl acetate 10/1, 5/1 or 3/1) on silica gel, obtaining the desired product.

#### Synthesis of 3-(1-(1*H*-indol-3-yl) dodecyl)-1*H*-indole (**3e**)

A mixture of indole **1** (8.54 mmol) and dodecanal **2e** (10.25 mmol) was stirred for the period indicated (TLC) at 100 °C. After reaction, the crude mixture was washed with CH_2_Cl_2_ and filtered through celite. The concentrated filtrate was flash chromatografied (hexane/ethyl acetate 10/1) on silica gel, obtaining the desired product.

### 3.3. Biology

#### Cell Line and Culture

Rat FaO cell line was supplied by the Interlab Cell Line Collection (Servizio Biotecnologie, IST, Genova, Italy). FaO cells were maintained in Dulbecco’s Modified Eagle’s Medium (DMEM plus Glutamax I, Invitrogen S.r.l., Milano, Italy) supplemented with penicillin, streptomycin and 10% heat-inactivated fetal calf-serum (FCS) (Invitrogen) in a humidified atmosphere of 5% CO_2_/95% air, at 37 °C. Indole 3-carbinol (**I3C**) and compound **3a**, **3b**, **4a** and **4b** were dissolved in DMSO and were added to the culture media to the final concentrations specified in the text. Control cells were treated with an equivalent amount of the solvent alone.

#### Cell Viability

Cell viability was determined by the uptake of neutral red by lysosome of viable cells. Determination of viability of adherent cells by NRU assay was performed according to Borefreund and Puerner [40]. The value obtained for treated cells was expressed as percentage of the value obtained in control cells. All experiments were performed in triplicate.

#### Statistical Analysis

Instant software (GraphpAd Prism 5, GraphPad Software Inc., San Diego, CA, USA) was used to analyse data. One-way analysis of variance (ANOVA) with post hoc analysis using Tukey’s multiple comparison test was used for parametric data. The results of multiple observations were presented as the means ± S.D. of three experiments. A *p* value of <0.05 was considered statistically significant.

## 4. Conclusions

We succeeded in the synthesis of new the 1-[1-(1*H*-indol-3-yl) alkyl]-1*H*-indoles by a simple and eco-friendly method, also assuming that a 2-azafulvene intermediate **6** could represent the key to the reaction mechanism. Moreover, CaO was demonstrated to be a valid alternative to organolithium reagents for the formation of indole-1-carbinol **5**, a potential building-block for biologically active compounds. Work continues on the improvement of the yields and on the development of new regioselective and stereoselective approaches to 1,3′-BIM isomers. Besides, we demonstrated that BIM derivatives **3b**, **4a** and **4b** show a behaviour similar to **3a** and are more potent than **I3C** in inducing loss of viability in hepatoma cells FaO, suggesting the possible employment of these compounds for therapeutic purposes, including liver injury and the selective elimination of tumoral liver cells. However, further studies are needed to elucidate the possible underlying mechanisms in action and their effects on the expression of some proteins involved in both Akt pathway-mediated oncogenic signaling, possibly leading to changes in the functional status of diverse effects or involved in cell cycle progression and apoptosis.

## Figures and Tables

**Table 1:** Base effect on reaction between indole **1** and formaldehyde **2a** at 100 °C.

Entry	Base	Base/(1)	Time (h)	Yields (%)	
				3a	4a
1	-	-	6	N.I. ^a^	N.I. ^a^
2	CaO	0.1/1	6	N.I. ^a^	N.I. ^a^
3	CaO	1/1	6	50	8
4	CaO	17/1	3	65	25
5	CaO ^b^	17/1	3	N.I. ^a^	62
6	KOH	1/1	1.5	61	37

^a^ Not Isolated. GC yields; ^b^ DMSO was used as solvent.

**Table 2:** Reaction of indole **1** with aliphatic and aromatic aldehydes. ^a^

Entry	Substrate	Base	Time (h)	Products		Yields (%) [ref.]	
				3	4	3	4
1	CH_3_CH_2_CHO **2b**	CaO	5	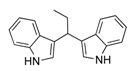	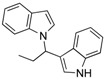	5	N.I. ^b^
2	CH_3_CH_2_CHO **2b**	-	3.5			57 [32]	38
3	CH_3_(CH_2_)_4_CHO **2c**	-	3.5	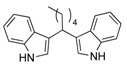	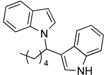	43 [32]	34
4	CH_3_(CH_2_)_5_CHO **2d**	-	3.5	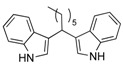	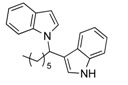	45 [33]	22
5	CH_3_(CH_2_)_10_CHO **2e**	-	3.5	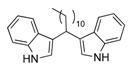	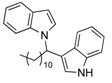	10	5
6	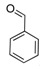 **2f**	-	3.5	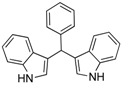	-	70 [32]	-
7	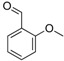 **2g**	-	3.5	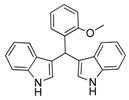	-	50 [9]	-
8	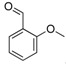 **2g**	CaO	5		-	N.I. ^b^	-
9	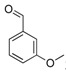 **2h**	-	3.5	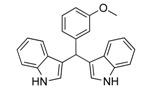	-	10 [13]	-
10	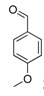 **2j**	-	3.5	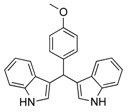	-	5 [32]	-
11	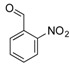 **2k**	-	3.5	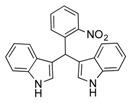	-	70 [34]	-
12	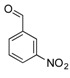 **2l**	-	3.5	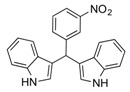	-	N.I. ^b^ [34]	-
13	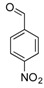 **2m**	-	3.5	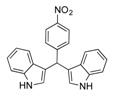	-	N.I.^b^ [32]	-
14	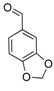 **2n**	-	3.5	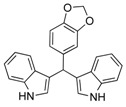	-	5 [8]	-
15	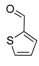 **2o**	-	3.5	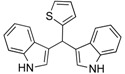	-	73 [34]	-
16	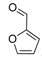 **2p**	-	3.5	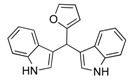	-	88 [32]	-

^a^ Operative temperature: 100 °C. All the reactions did not occur at room temperature; ^b^ Not Isolated. GC yields.

## Supplementary Materials

Compound characterization and NMR spectra associated with this article are available online.
