# Supplementary material for: Solvent-Free Addition of Indole to Aldehydes: Unexpected Synthesis of Novel 1-[1-(1H-Indol-3-yl) Alkyl]-1H-Indoles and Preliminary Evaluation of Their Cytotoxicity in Hepatocarcinoma Cells
Source: Molecules. 2017 Oct 17;22(10):1747. doi: 10.3390/molecules22101747 (PMC6151463; doi:10.3390/molecules22101747)
Supplement: Supplementary file 1 [file molecules-22-01747-s001.pdf]

Solvent Free Addition of Indole to Aldehydes: Unexpected Synthesis of Novel 1-[1-(1H-Indol-3-yl) Alkyl]-1H-Indoles and Preliminary Evaluation of Their Cytotoxicity on Hepatocarcinoma Cells

Graziella Tocco [1,*], Gloria Zedda [2], Mariano Casu [3], Gabriella Simbula [4,†], Michela Begala [5,†]

SUPPORTING INFORMATION

Characterization of novel compounds and NMR spectra.

COMPOUNDS CHARACTERIZATION

Synthesis of 1-[1-(1H-indol-3-yl) methyl]-1H-indole (4a).

Yield: 62%. Oil.

IR (neat): 3080, 1510, 1420, 1220 cm^{-1}.

^{1}H NMR (400 MHz, DMSO): 11.15 (bs, 1H), 7.77 (d, ^3J = 8.0 Hz, 1H), 7.64 (d, ^3J =3.2 Hz, 1H), 7.63 (d, ^3J = 8.0 Hz, 1H), 7.62 (d, ^3J = 7.2, 1H), 7.60 (s, 1H), 7.46 (d, ^3J = 8.0 Hz, 1H), 7.21 (t, ^{3}J = 8.0 Hz, 1H), 7.17 (t, ^{3}J = 8.0, 1H), 7.09 (t, ^{3}J = 8.0 Hz, 1H), 7.03 (t, ^{3}J = 7.2 Hz, 1H), 6.51 (d, ^3J = 3.2 Hz, 1H), 5.63 (s, 2H) ppm. ^{13}C NMR (300 MHz, DMSO): δ 136.39, 135.70, 128.87, 128.33, 126.37, 124.88, 121.30, 120.85, 120.42, 118.83, 118.60, 118,52, 116,40, 111.04, 110.28, 100.33, 41.33 ppm. EI-MS m/z: 246 (100), 230 (3), 218 (5), 130 (90), 117 (78), 103 (19), 89 (23), 77 (21), 63 (17), 51 (7). HRMS Calcd for $C_{17}H_{15}N_2$ $[M+H]^+$: 247.1235. Found: 247.1226.

1-[1-(1H-indol-3-yl) propyl]-1H-indoles (4b).

Yield: 38%. Viscous liquid.

IR (neat): 3100, 2850, 1580, 1470, 1230 cm^{-1}.

^{1}H NMR (400 MHz, DMSO): 11.17 (bs, 1H), 7.80 (d, ^{3}J = 8.0 Hz, 1H), 7.66 (d, ^{3}J = 2.0 Hz, 1H), 7.62 (d, ^{3}J = 7.9 Hz, 1H), 7.56 (d, ^{3}J = 7.9 Hz, 1H), 7.44 (d, ^{3}J = 8.0 Hz, 1H), 7.32 (d, ^{3}J = 8.0 Hz, 1H), 7.20 (t, ^{3}J = 8.0 Hz, 1H), 7.13 (t, ^{3}J = 8.0 Hz, 1H), 7.09 (t, ^{3}J = 7.9 Hz, 1H), 6.94 (t, ^{3}J = 8.0 Hz, 1H), 6.52 (d, ^{3}J = 7.9 Hz, 1H), 5.92 (t, ^{3}J = 7.3 Hz, 1H), 2.52 (q, 2 H), 0.93 (t, ^{3}J = 7.3 Hz, 3H) ppm. ^{13}C NMR (400 MHz, DMSO): δ 136.48, 136.07, 128.21,126.62, 126.23, 123.30, 121.25, 120.95, 120.46, 118.90, 118.71, 118.51, 115.30, 111.48, 110.31, 100.74, 53.88, 27.62, 11.39 ppm. EI-MS: m/z: 274 (5), 258 (1), 245 (4), 158 (100), 143 (8), 130 (12), 117 (25), 89 (7). HRMS Calcd for $C_{19}H_{18}N_2$ $[M+H]^+$: 275.1548. Found: 275.1547.

1-[1-(1H-indol-3-yl) hexyl]-1H-indoles (4c).

Yield: 34%. Sticky oil.

IR (neat): 3100, 2760, 1600, 1510, 1300 cm^{-1}.

1H NMR (400 MHz, DMSO): 11.10 (bs, 1H), 7.88 (d, ^3J = 7.8 Hz, 1H), 7.76 (d, ^3J = 2.0 Hz, 1H), 7.60 (d, ^3J = 8.0 Hz, 1H), 7.54 (d, ^3J = 8.0 Hz, 1H), 7.48 (d, ^3J = 7.8 Hz, 1H), 7.33 (d, ^3J = 7.8 Hz, 1H), 7.24 (t, ^3J = 7.8 Hz, 1H), 7.17 (t, ^3J = 7.8 Hz, 1H), 7.09 (t, ^3J = 8.0 Hz, 1H), 7.00 (t, ^3J = 7.8 Hz, 1H), 6.58 (d, ^3J = 8.0 Hz, 1H), 5.64 (t, ^3J = 7.2 Hz, 1H), 2.23 (q, 2 H), 1.99 (m, 4H), 1.55 (quint, 2H), 0.96 (t, ^3J = 7.2 Hz, 3H) ppm. ^{13}C NMR (400 MHz, DMSO): δ 135.22, 134.00, 127.81, 126.59, 124.23, 123.78, 121.29, 121.48, 120.87, 118.44, 118.13, 117.51, 115.22, 110.78, 110.35, 100.67, 59.80, 37.41, 32.54, 27.18, 25.98, 12.87 ppm. EI-MS m/z: 316 (2), 245 (4), 218 (1), 200 (100), 156 (4), 130 (23), 117 (8). Anal. Calcd for $C_{22}H_{24}N_2$: C 83.54, H 7.59, N 8.86. Found: C 83.51, H 7.63, N 8,85.

1-[1-(1H-indol-3-yl) heptyl]-1H-indoles (4d).

Yield: 22%. Sticky oil.

IR (neat): 3200, 2790, 1620, 1460, 1200 cm^{-1}.

^1H NMR (400 MHz, DMSO): 11.21 (bs, 1H), 7.75 (d, ^3J = 8.0 Hz, 1H), 7.64 (d, ^3J = 1.8 Hz, 1H), 7.57 (d, ^3J = 7.6 Hz, 1H), 7.52 (d, ^3J = 7.6 Hz, 1H), 7.48 (d, ^3J = 8.0 Hz, 1H), 7.37 (d, ^3J = 8.0 Hz, 1H), 7.29 (t, ^3J = 8.0 Hz, 1H), 7.14 (t, ^3J = 8.0 Hz, 1H), 7.09 (t, ^3J = 7.6 Hz, 1H), 6.96 (t, ^3J = 8.0 Hz, 1H), 6.68 (d, ^3J = 7.6 Hz, 1H), 5.21 (t, ^3J = 7.3 Hz, 1H), 2.98 (q, 2 H), 2.09 (m, 6H), 1.87 (quint, 2H), 0.89 (t, ^3J = 7.3 Hz, 3H) ppm ^{13}C NMR (300 MHz, DMSO): δ 138.78, 136.05, 127.99, 126.67, 124.00, 123.54, 121.34, 121.18, 120.69, 119.44, 118.24, 117.52, 115.29, 111.78, 110.99, 100.96, 60.00, 37.56, 32.45, 29.41, 27.17, 25.88, 11.99 ppm. EI-MS m/z: 330 (3), 245 (5), 214 (100), 156 (3), 143 (3), 130 (72), 117 (22), 103 (2), 89 (3). Anal. Calcd for $C_{23}H_{26}N_2$: C 83.64, H 7.88, N 8.48. Found: C 83.60, H 7.91, N 8,46.

1-[1-(1H-indol-3-yl) dodecyl]-1H-indoles (4e).

Yield: 5%. Sticky oil.

IR (neat): 3180, 2930, 1620, 1450, 1220 cm^{-1}.

^1H NMR (400 MHz, DMSO): 11.19 (bs, 1H), 7.72 (d, ^3J = 7.9 Hz, 1H), 7.65 (d, ^3J = 2.0 Hz, 1H), 7.63 (d, ^3J = 8.2 Hz, 1H), 7.58 (d, ^3J = 8.2 Hz, 1H), 7.53 (d, ^3J = 7.9 Hz, 1H), 7.39 (d, ^3J = 7.9 Hz, 1H), 7.26 (t, ^3J = 7.9 Hz 1H), 7.19 (t, ^3J = 7.9 Hz 1H), 7.13 (t, ^3J = 8.2 Hz,1H), 6.97 (t, ^3J = 7.9 Hz 1H), 6.68 (d, ^3J = 8.2 Hz, 1H), 5.77 (t, ^3J = 7.2 Hz,1H), 2.83 (q, 2 H), 1.67 (m, 6 H), 1.55 (m, 8 H), 1.13 (m, 4 H), 0.75 (t, ^3J = 6.9 Hz,3H) ppm.

^{13}C NMR (300 MHz, DMSO): δ 137.29, 134.98, 127.65, 126.59, 124.51, 123.88, 121.27, 121.59, 120.86, 118.42, 118.22, 117.55, 115.27, 110.77, 110.35, 101.37, 59.34, 38.01, 32.94, 29.70, 29.67, 29.63, 29.61, 29.60, 29.57, 27.18, 25.78, 11.82 ppm.

EI-MS m/z: 400 (1), 285 (100), 245 (1), 207 (1), 156 (5), 130 (25), 117 (5).

Anal. Calcd for $C_{28}H_{36}N_2$: C 84.00, H 9.00, N 7.00. Found: C 83.96, H 9.04, N 6.98.

3-(1-(1H-indol-3-yl) dodecyl)-1H-indole (3e)

Yield: 10%. Oil.

IR (neat): 3190, 2760, 1630, 1470 cm^{-1}.

^{1}H NMR (300 MHz, $CDCl_3$): δ 7.92 (bs, 2H), 7.60 (d, ^{3}J = 7.8 Hz, 2H), 7.32 (d, ^{3}J = 7.8 Hz, 2H), 7.15 (t, ^{3}J = 7.8 Hz, 2H), 7.03 (t, ^{3}J = 7.8 Hz, 2H), 6.99 (s, 2H), 4.39 (t, ^{3}J = 7.3 Hz 1H), 2.45 (q, 2 H), 1.56 (m, 6 H), 1.38 (m, 8 H), 1.01 (m, 4 H), 0.85 (t, ^{3}J = 6.8 Hz 3H) ppm.

^{13}C NMR (300 MHz, $CDCl_3$): δ 136.57, 127.21, 121.68, 121.43, 120.30, 119.69, 118.94, 111.03, 44.38, 39.00, 32.79, 31.95, 30.05, 29.88, 29.71, 29.37, 28.69, 22.71, 18.14, 13.07 ppm.

EI-MS m/z: 400 (3), 218 (4), 156 (1), 130 (4), 117 (1).

Anal. Calcd for $C_{28}H_{36}N_2$: C 84.00, H 9.00, N 7.00. Found: C 84.02, H 8.91, N 6.94.

NMR SPECTRA

COSY (DMSO) Compound (4a)

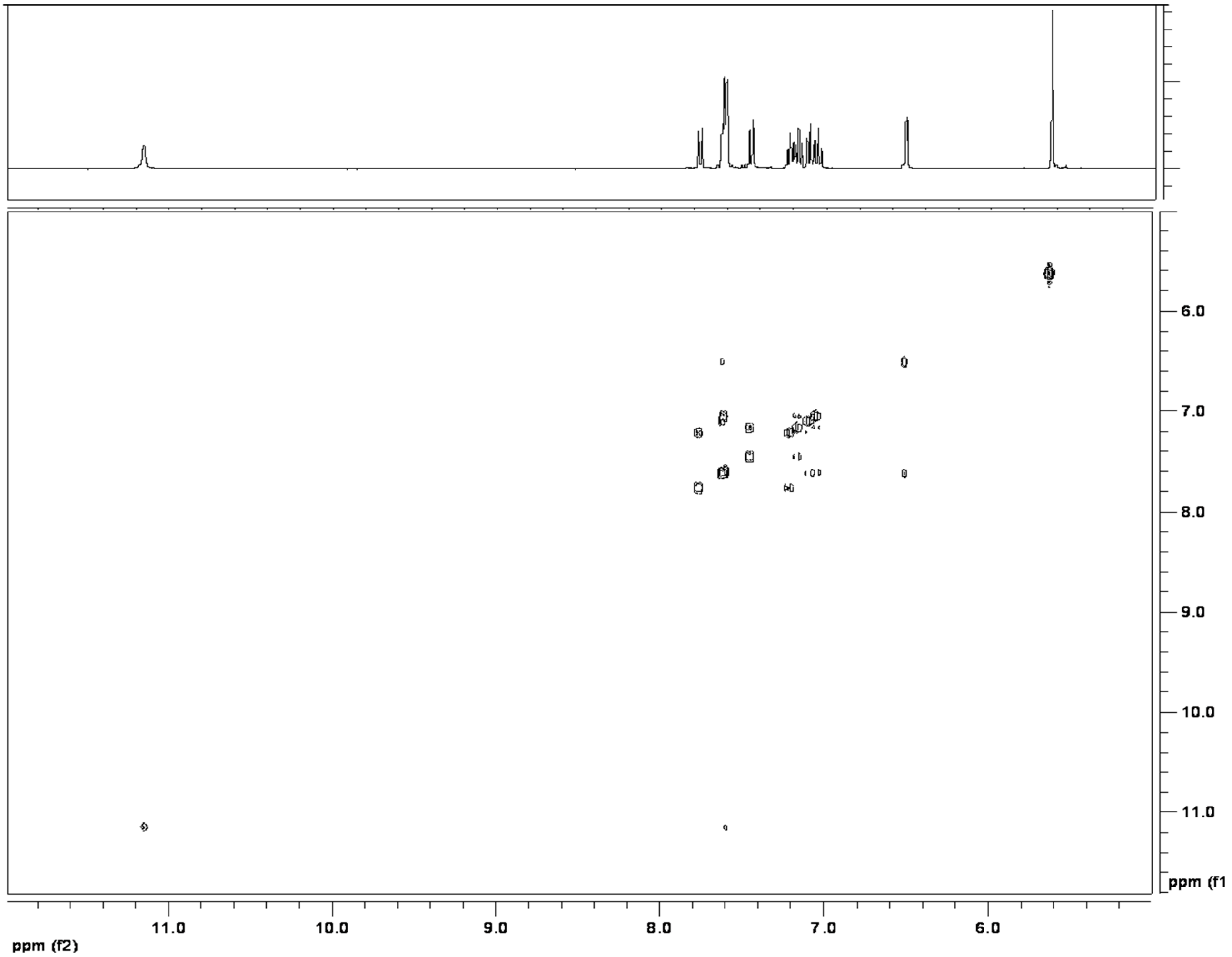

^{1}H NMR (DMSO) Compound (4a)

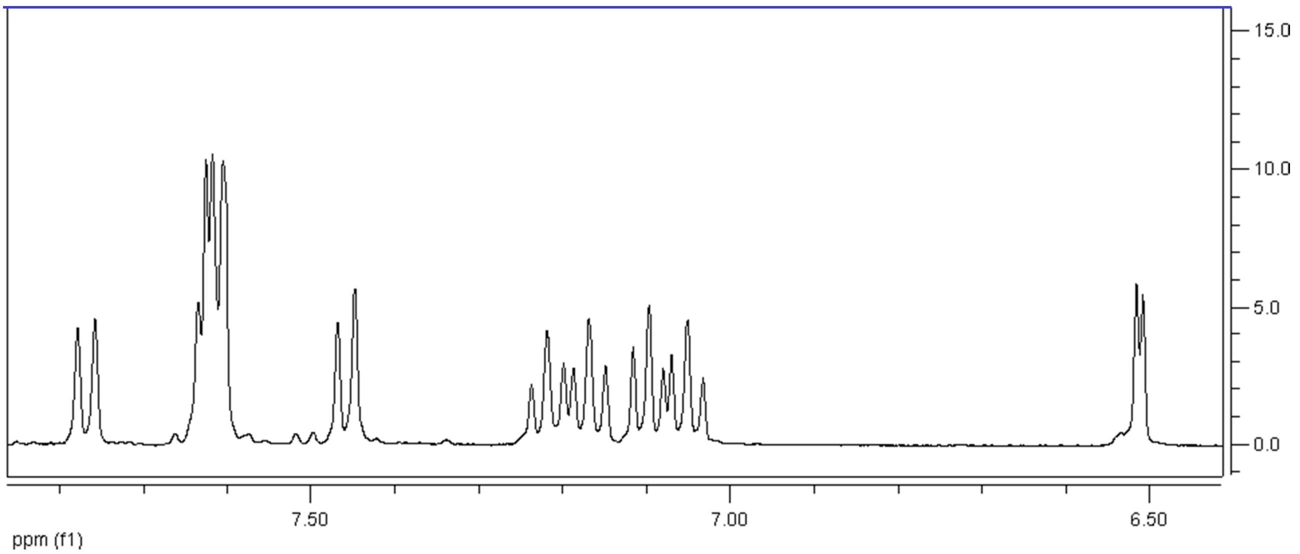

TOCSY (DMSO) Compound (4a)

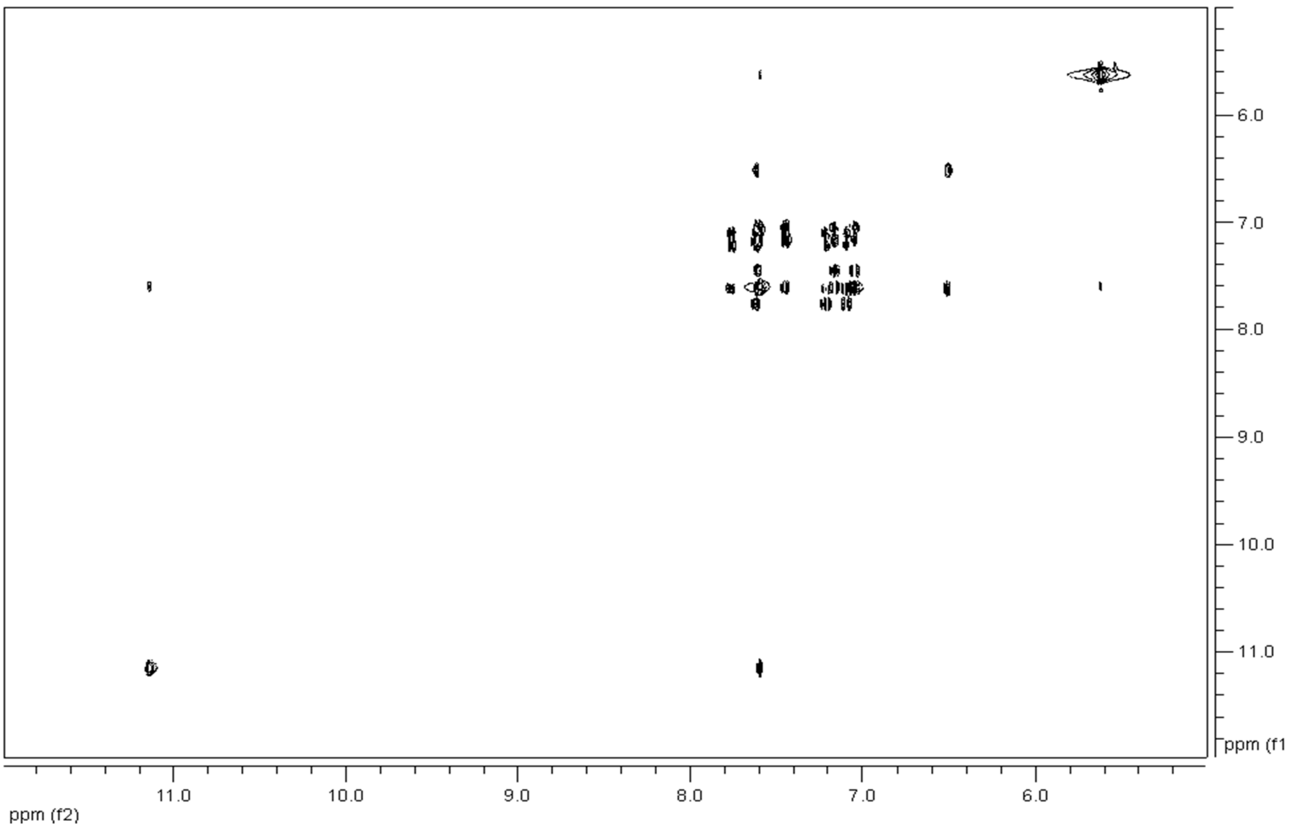

^{13}C NMR (DMSO) Compound (4a)

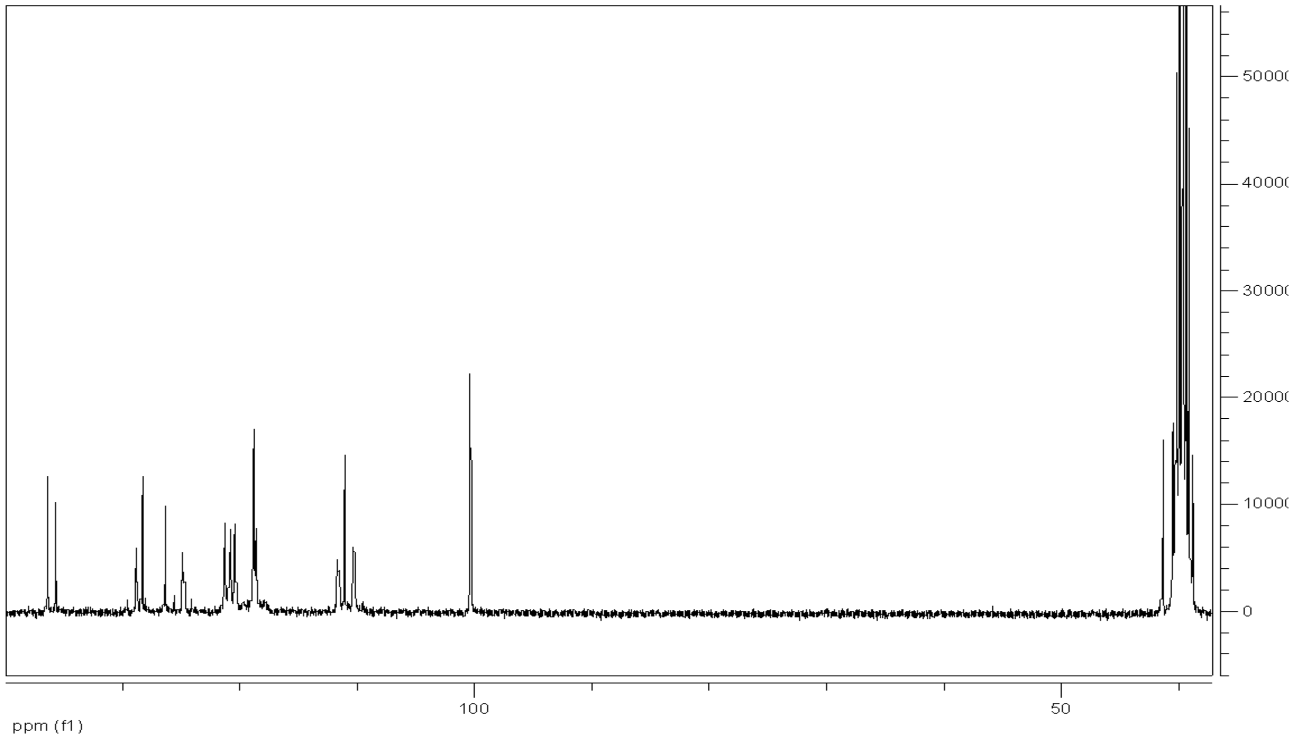

^{1}H NMR (DMSO) Compound (4b)

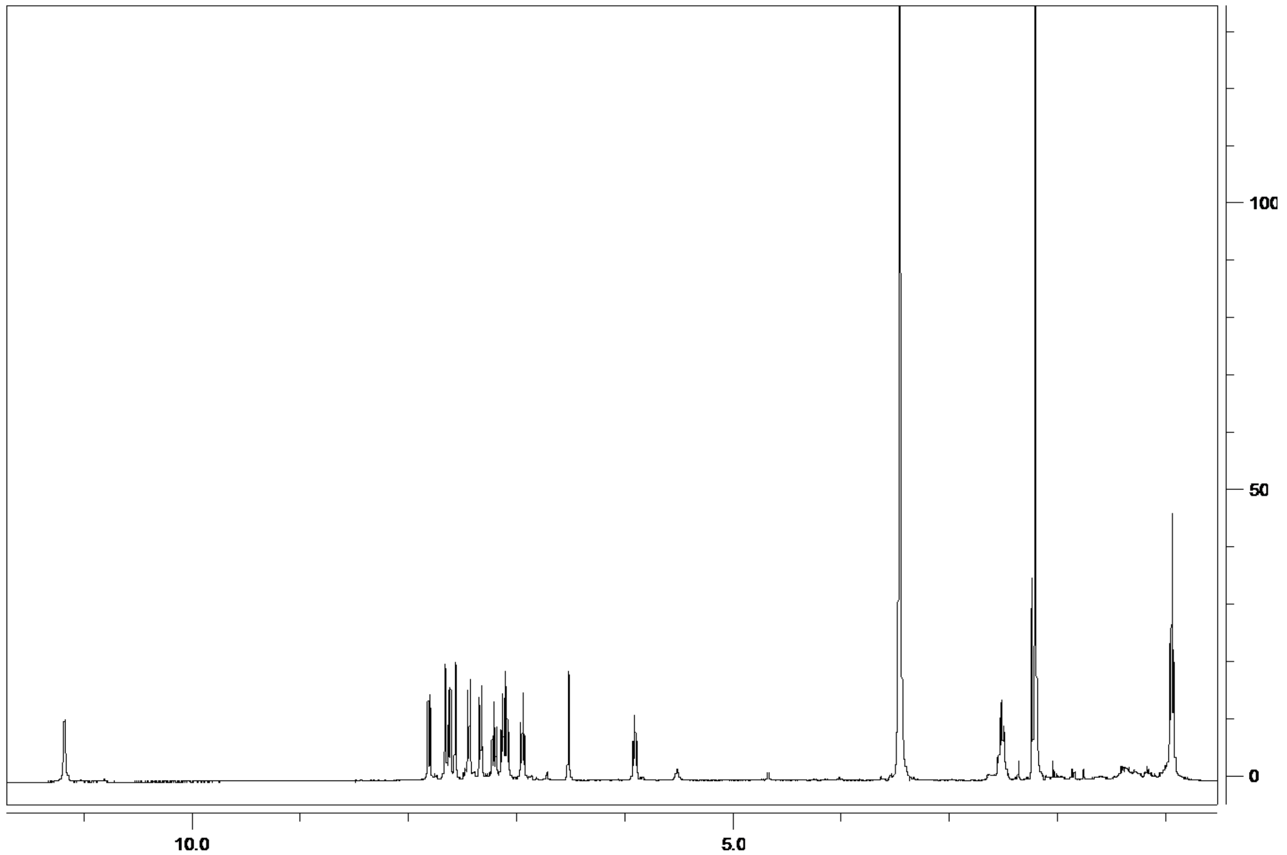

^{1}H NMR (DMSO) Compound (4b)

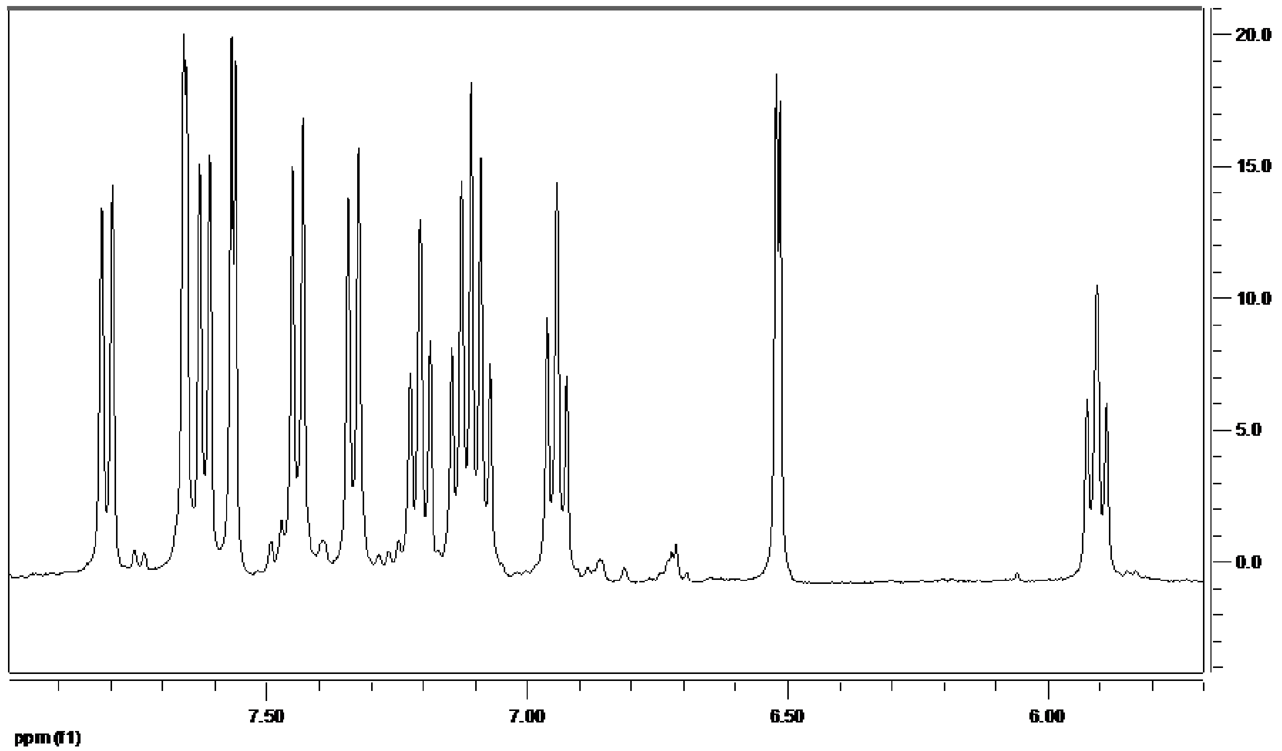

COSY (DMSO) Compound (4b)

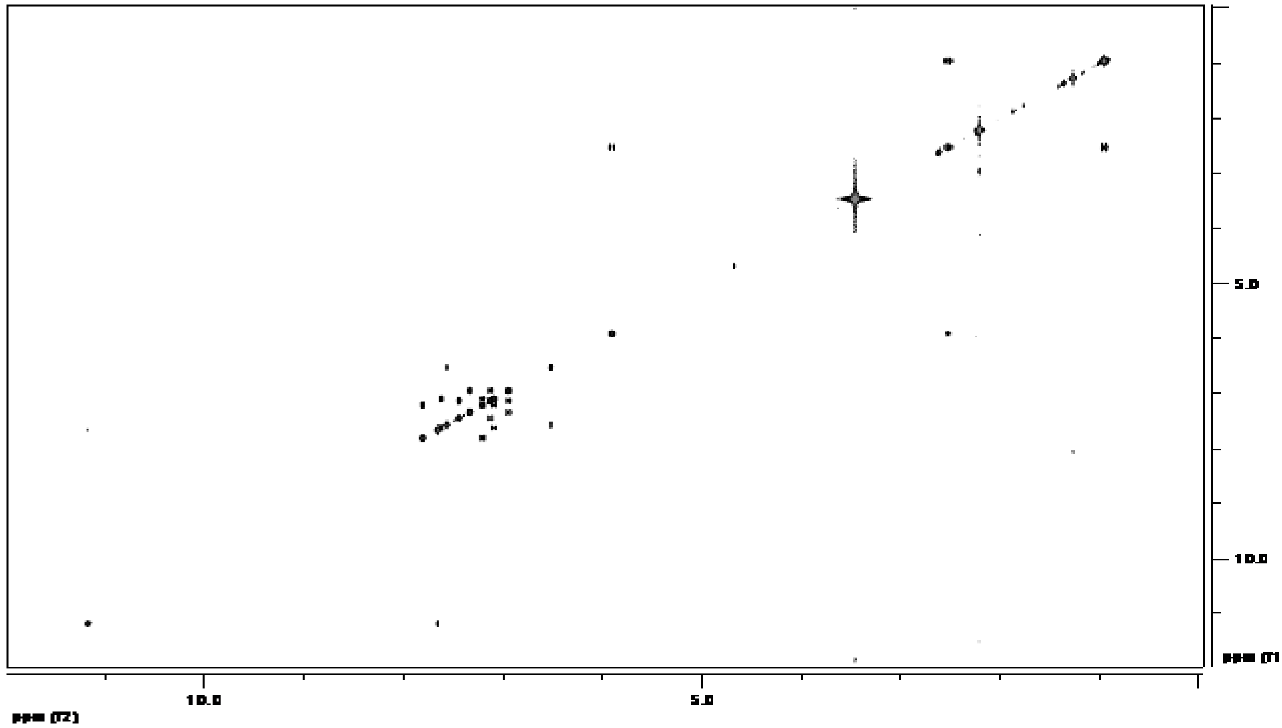

COSY (DMSO) Compound (4b)

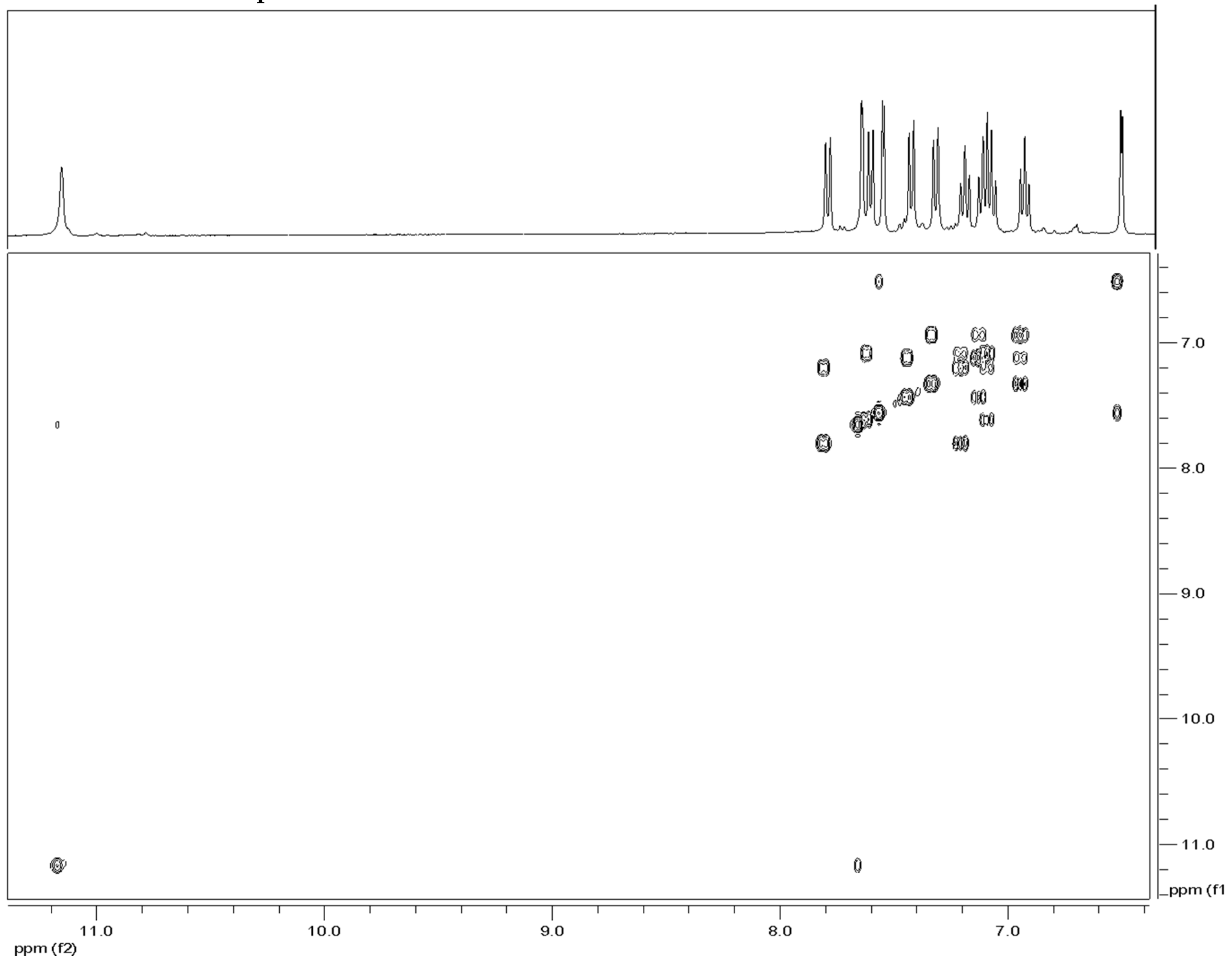

^{13}C NMR (DMSO) Compound (4b)

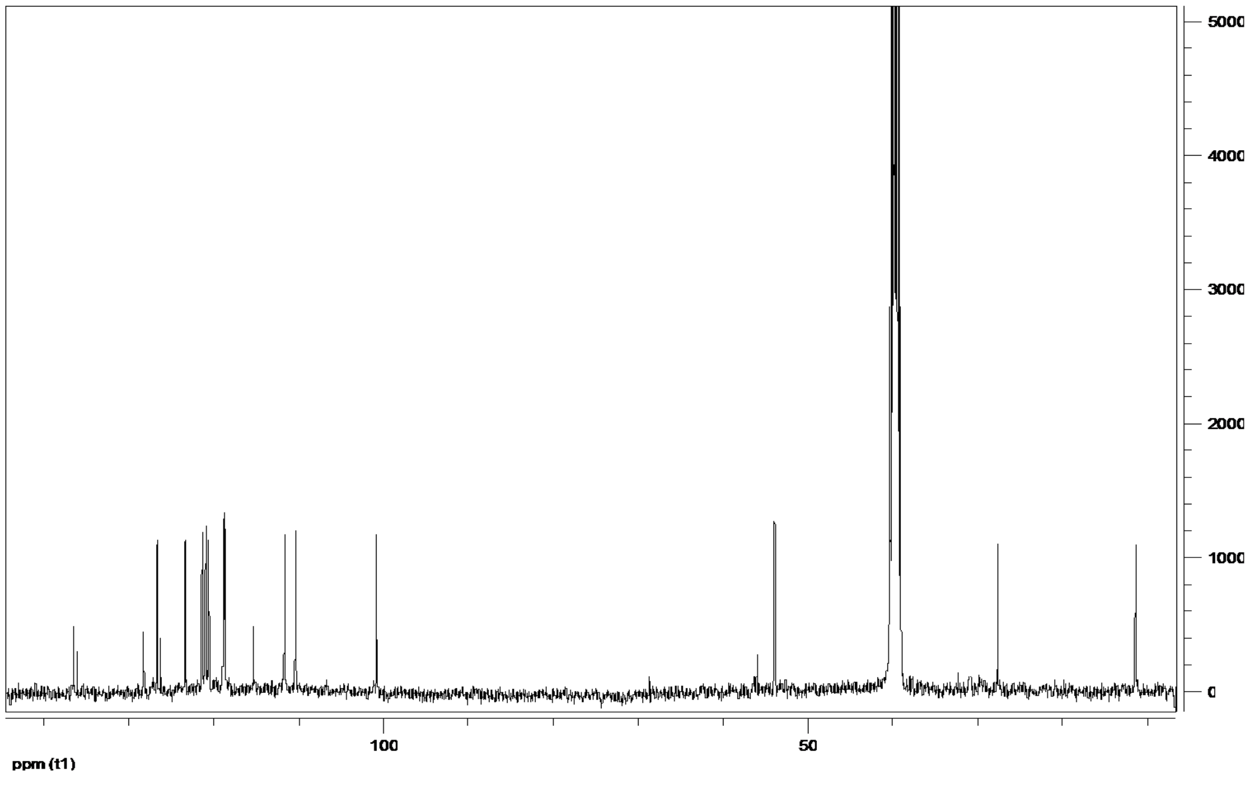

gHMQC (DMSO) Compound (4b)

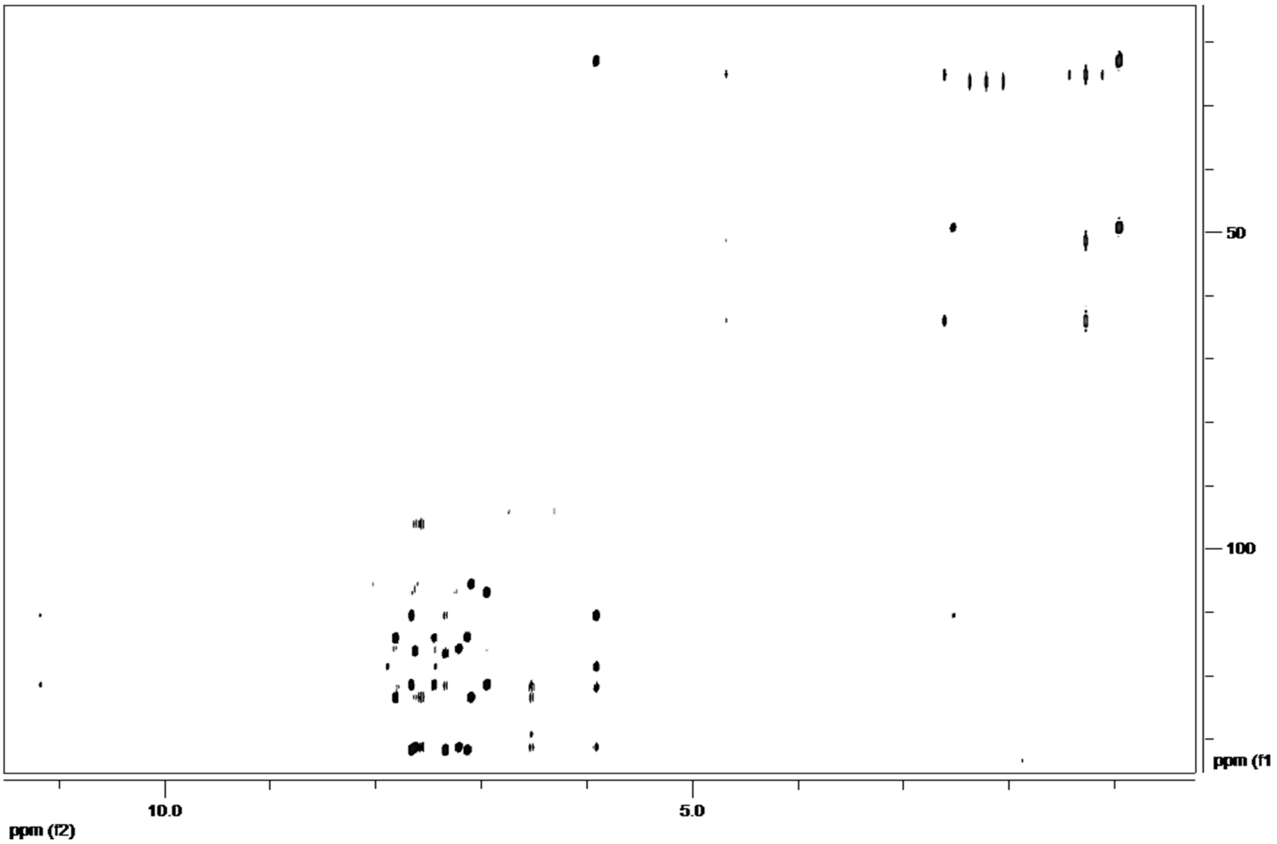

gHSQC (DMSO) Compound (4b)

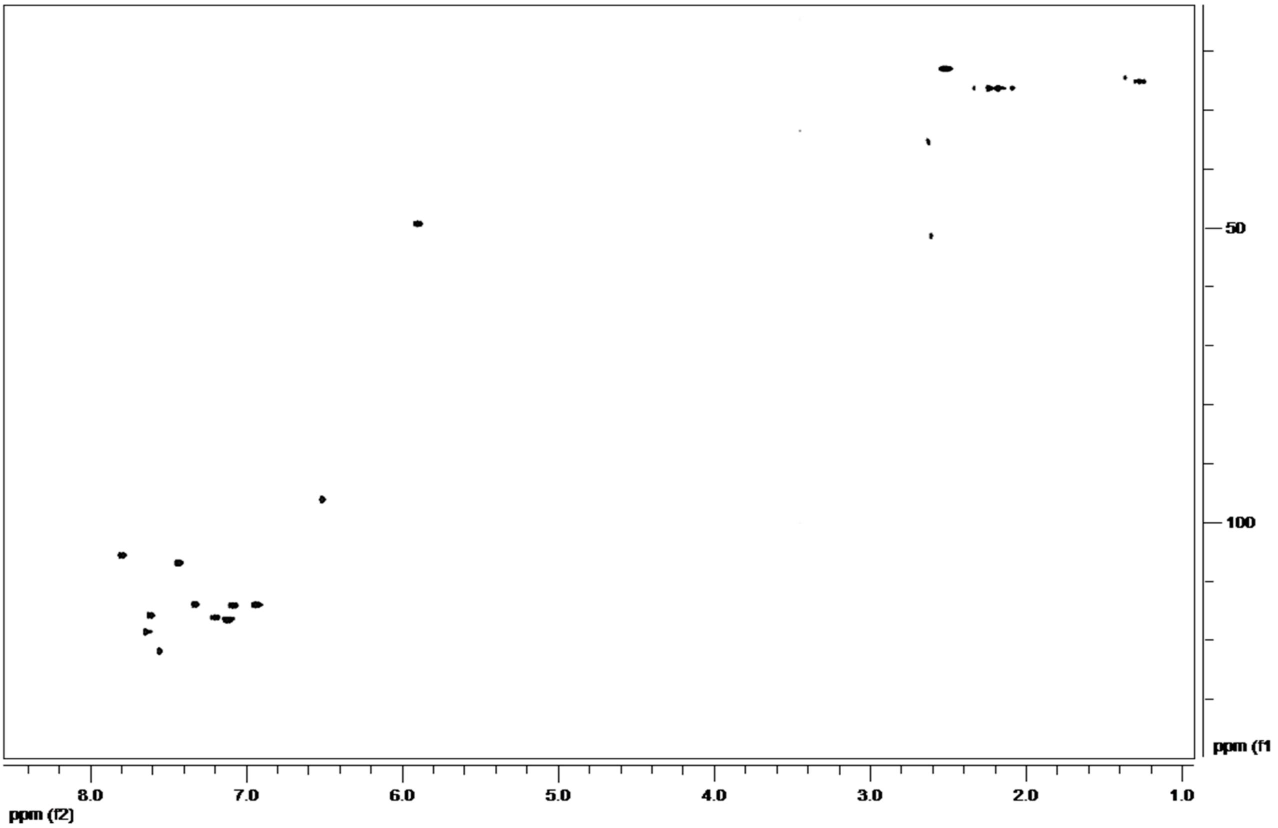

Table 1. ^{1}H, ^{13}C chemical shift and ^{1}H coupling constant obtained from ^{1}H, ^{13}C, COSY, gHSQC, gHMQC and ROESY spectra of compound **4b.**

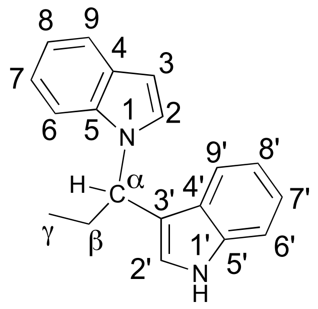

N°	δ_H mult (J Hz)	δ_C	HMQC
2	7.56, d ($J_{2,3}$=7.9)	126.62	C-4,5
3	6.52, d	100.74	C-4,8
4		128.21	
5		136.07	
6	7.80, d ($J_{6,7}$=8.0)	110.31	C-4,8
7	7.20, t	120.95	C-5,9
8	7.09, t	118.90	C-4,6
9	7.62, d ($J_{8,9}$=7.9)	120.46	C-7,5
2′	7.66, d ($J_{2',NH}$ =2.0)	123.30	C-3′,4′,5′
3′		115.30	
4′		126.23	
5′		136.48	
6′	7.44, d ($J_{6',7'}$=8.0)	111.48	C-4′
7′	7.13, t	121.25	C-5′9′
8′	6.94, t	118.71	C-4′,6′
9′	7.32, d ($J_{8',9'}$=8.0)	118.51	C-7′,5′
NH	11.17, s (broad)		
α	5.92, t (J , =7.3)	53.88	C-2′,3′,
β	2.52, quint	27.62	C-
γ	0.93, t ((J , =7.3)	11.39	C-
